# Multidrug-resistant *Proteus mirabilis* in a critically endangered Malayan pangolin: clinical and genomic insights

**DOI:** 10.3389/fvets.2025.1552499

**Published:** 2025-04-30

**Authors:** Ziqiao Chen, Jiayi Wang, Kai Wang, Fuyu An, Shasha Liu, Haikuo Yan, Yan Hua

**Affiliations:** ^1^College of Veterinary Medicine, South China Agricultural University, Guangzhou, China; ^2^Guangdong Provincial Key Laboratory of Silviculture, Protection and Utilization, Guangdong Academy of Forestry, Guangzhou, China

**Keywords:** *Proteus mirabilis*, Malayan pangolin, antibiotic resistance, pathogenicity, multidrug resistance

## Abstract

*Proteus mirabilis*, an important zoonotic opportunistic pathogen, is widely found in nature and the intestinal tracts of animals, which can cause diarrhea, pneumonia, urinary tract infections, and other symptoms in domestic animals including sheep, pigs, cattle and chickens. In this study, necropsy of a deceased critically endangered Malayan pangolin revealed lobar pneumonia in the lungs and hepatocyte necrosis with hepatic cord disintegration in the liver. A strain of *Proteus mirabilis* (PM2022) was isolated from the affected lungs and liver. This bacterium exhibited multidrug resistance, being susceptible only to cefoxitin and amikacin. Whole-genome sequencing identified 26 antibiotic resistance genes, including *CTX-M-65*, *FosA3*, which mediate resistance to five classes of antibiotics, such as penicillins and quinolones. Additionally, 20 virulence factors (including the T6SS secretion system, hemolysins HpmA/B, among others) were detected. Mouse experiments confirmed its high pathogenicity (LD_50_ = 1.45 × 10^9^ CFU/mL). Based on experimental and genomic testing results, the initial symptoms of *Proteus mirabilis* infection in pangolins manifest in the lungs, liver, and intestines, and the use of penicillins and quinolones should be avoided during treatment. This study offers clinical guidance for diagnosing and treating *P. mirabilis* infections in pangolins, informing evidence-based antimicrobial strategies.

## 1 Introduction

Malayan pangolin (*Manis javanica*), a critically endangered mammal (IUCN Red List 2023), primarily inhabits primary and tropical rainforests in Southeast Asia (1). As one of the most heavily trafficked mammals globally, pangolins face severe population declines due to illegal hunting for their scales (used in traditional medicine) and meat (2, 3). Conservation efforts, including rescue programs and captive breeding, are critical for population recovery. However, the current success rate of pangolin rehabilitation remains low, with bacterial infections being a significant cause of mortality in rescued individuals (4). *Proteus mirabilis* is a gram-negative bacillus belonging to the Morganellaceae family, which can differentiate from a short rod into an elongated multinucleated cell form expressing thousands of flagella (5). *P. mirabilis* is an important opportunistic zoonotic pathogen (6), widely distributed in nature and in the intestines of animals. In recent years, *P. mirabilis* has been recognized as a reservoir of virulence genes and antibiotic resistance genes (ARGs) (7, 8). The high prevalence of multidrug-resistant (MDR) strains of *P. mirabilis* has become an increasingly serious public health issue, posing significant threats to both wildlife and humans (9). In clinical settings, it has garnered significant attention as one of the most common causative agents of urinary tract infections (UTIs) (10). It can cause diarrhea, pneumonia, UTIs, and conjunctivitis in various animals, including sheep (11), pigs (12), cattle (13, 14), and chickens (15). Infections have also been reported in wild animals such as South China tigers (16), giant pandas (17), and red pandas (18), leading to diarrhea, abortion, septicemia, and other life-threatening conditions. Its multidrug resistance further complicates treatment. In the management of captive pangolins, bacterial infections are one of the primary causes of disease and mortality (4). Previous studies have shown that *P. mirabilis* is an important pathogen causing symptoms such as diarrhea (19), nephritis (20), pulmonary hemorrhage, and hepatic congestion (21) in Malayan pangolins. However, the mechanisms of antibiotic resistance and pathogenicity of *P. mirabilis* isolated from pangolins have not been fully clarified, and there are no comprehensive reports analyzing its resistance and virulence genes. This study isolated and cultured *P. mirabilis* from the liver and lungs of a deceased Malayan pangolin. By characterizing its antibiotic resistance, virulence, and pathogenicity, this research aims to improve the clinical management of bacterial infections in pangolins. These findings will enable more effective medical treatments, boosting rescue success and captive breeding efforts, which are vital for saving this critically endangered species from extinction.

## 2 Methods

### 2.1 Animal and collection of samples

In 2022, a Malayan pangolin that was transferred to the Guangdong Provincial Wildlife Rescue Monitoring Center died. A necropsy was performed to examine the lesions. Under sterile conditions, scalpels were used to excise sections from the affected areas of the liver and lungs. Tissue samples were collected using forceps and evenly spread onto Blood Agar plates (Haibo Biology, Qingdao, China). The plates were then incubated at 37°C for 24 h in a microbial incubator (Yi Heng, Shanghai, China). Dominant colonies were selected and subcultured onto Brain Heart Infusion Broth (BHI) Agar plates (Haibo Biology, Qingdao, China) to purify them into single colonies. The morphology of the colonies was observed, and Gram staining was performed (Beckman, China). The staining characteristics and morphology of the bacterial cells were examined under a microscope (100 × magnification).

### 2.2 Identification of isolates

The isolated strains were inoculated onto Triple Sugar Iron (TSI) slant medium and cultured overnight at 37°C. Colonies were then picked and inoculated into a biochemical identification kit for *Proteus* species (Haibo Biology, Qingdao, China). The results were interpreted according to the diagnostic criteria and handling principles for *Proteus* food poisoning as outlined in the Health Industry Standard of the People’s Republic of China (WS/T9-1996).

Genomic DNA of the bacterial isolate was extracted according to the manufacturer’s instructions (Tiangen, Beijing, China) and used as a template for PCR (22). The 16S rRNA gene was amplified using primers (5′-AGAGTTTGATCMTGGCTCAG-3′ and 5′-GGTTACCTTGTTACGACTT-3′). The PCR positive products were sequenced by Sangon Biotech (Shanghai).

### 2.3 Phylogenetic tree analysis

The 16S rRNA gene sequence of the strain was compared with reference sequences in GenBank using BLAST analysis. Highly homologous sequences were downloaded and aligned using MEGA 11.0 software. A phylogenetic tree was constructed using the maximum likelihood method.

### 2.4 Antimicrobial susceptibility assay

The antimicrobial susceptibility of the isolated strain was determined using the Kirby-Bauer (K-B) disk diffusion method. The bacterial solution of the strain was evenly spread on Mueller-Hinton (MH) agar plates (Haibo, Qingdao, China), and antibiotic disks were placed. The inhibition zone diameter was measured after incubation at 37°C for 18 h. *E. coli* ATCC 25922 was used as the quality control strain. The study evaluated 35 commonly used antibiotics (Hangzhou Microbial, China), including: penicillin (PEN, 1 μg), ampicillin (AMP, 10 μg), oxacillin (OXA, 1 μg), amoxicillin (AMX, 25 μg), cephalexin (LEX, 30 μg), cefoxitin (FOX, 30 μg), cefepime (CFP, 30 μg), ceftiofur (TIO, 30 μg), ceftazidime (CAZ, 30 μg), ceftriaxone (CRO, 30 μg), cefotaxime (CTX, 30 μg), meropenem (MEN, 10 μg), imipenem (IPM, 10 μg), gentamicin (GEN, 10 μg), neomycin (NEO, 30 μg), kanamycin (KAN, 30 μg), tobramycin (TOB, 10 μg), amikacin (AMK, 30 μg), erythromycin (ERY, 15 μg), azithromycin (AZM, 15 μg), clindamycin (CLI, 2 μg), norfloxacin (NOR, 10 μg), levofloxacin (LEV, 5 μg), enrofloxacin (ENR, 10 μg), tetracycline (TET, 30 μg), doxycycline (DOX, 30 μg), florfenicol (FFC, 30 μg), chloramphenicol (CHL, 30 μg), fusidic acid (FUS, 10 μg), trimethoprim-sulfamethoxazole (SXT, 20 μg), polymyxin B (PB, 300 μg), nitrofurantoin (NIT, 300 μg), rifampin (RIF, 5 μg), and vancomycin (VAN, 30 μg). Susceptibility was interpreted according to the CLSI VET standards (2023 version) (23).

### 2.5 Median lethal dose determination

The median lethal dose (LD_50_) was determined using the Kärber method (24). SPF mice (20-day-old Kunming strain, equal males/females) were intraperitoneally injected with bacterial suspensions at concentrations of 2.9 × 10^7^, 2.9 × 10^8^, 2.9 × 10^9^, 2.9 × 10^10^, 2.9 × 10^11^ CFU/mL, respectively. Each group consisted of 10 mice. The experimental group was intraperitoneally injected with 0.2 mL of bacterial suspension, while the controls received sterile saline. Mortality was recorded over a one-week period, and deceased mice underwent necropsy and pathogen re-isolation.

### 2.6 Whole-genome sequencing and bioinformatics

DNA of the isolate was extracted to construct libraries, followed by cluster preparation and sequencing. The total DNA of the sample was sequenced using the Illumina second-generation high-throughput sequencing platform (25). Subsequently, the gene functions were annotated. The ARGs and virulence factors were predicted using the Comprehensive Antibiotic Resistance Database (CARD) and the Virulence Factors of Pathogenic Bacteria database (VFDB), respectively.

## 3 Results

### 3.1 Necropsy and histopathological findings

**Figure 1:**
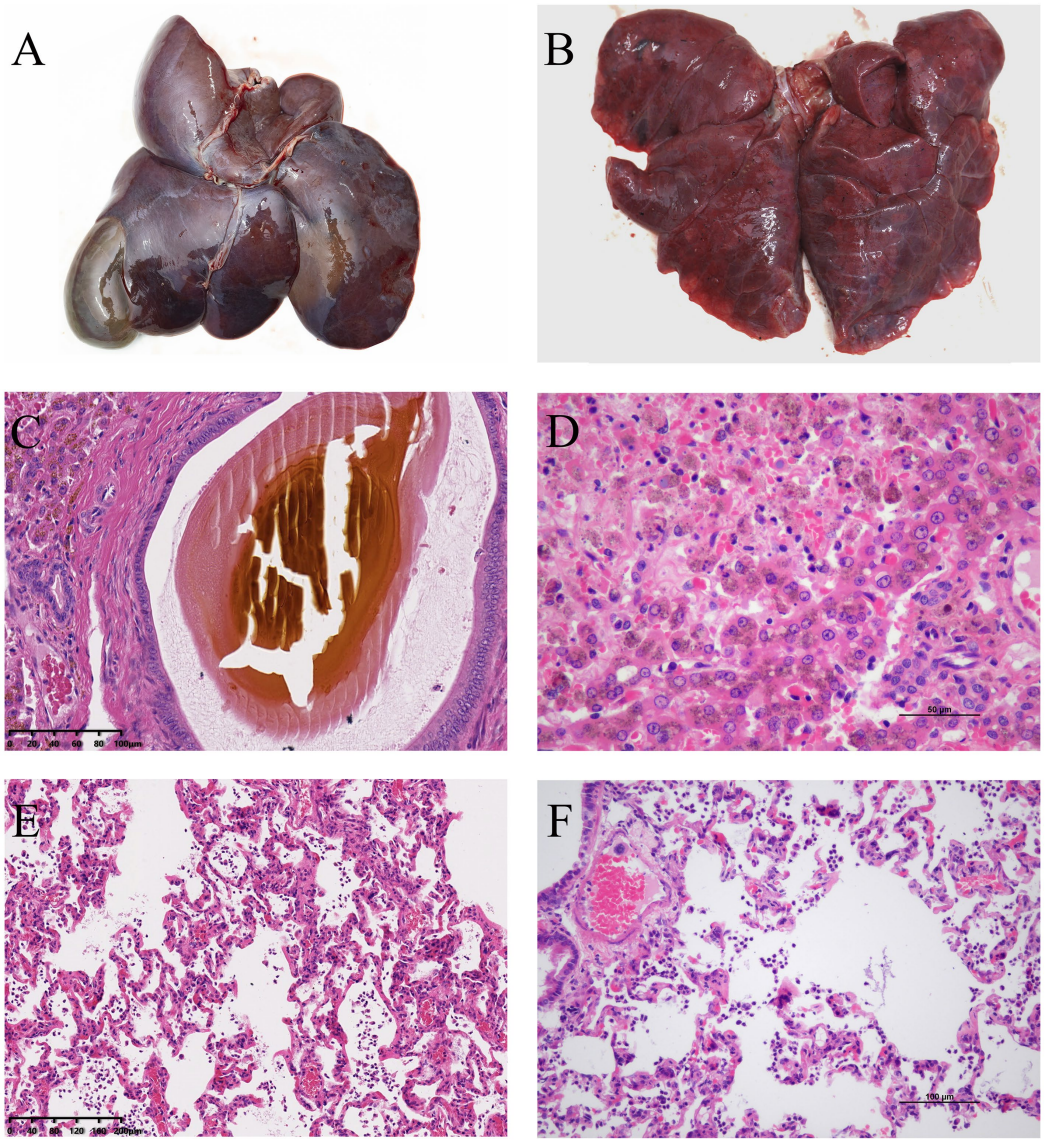
**(A)** Necropsy revealed a dark red liver with enlarged gallbladder. **(B)** The lungs showed localized necrotic foci. **(C)** H&E staining showed bilirubin crystals within the bile ducts of the liver. **(D)** Extensive necrosis was observed in the liver parenchyma, with hepatocyte necrosis, disintegration of hepatic cords, congestion of hepatic sinusoids, and significant pigment deposition in the cytoplasm of hepatocytes. **(E,F)** The lungs exhibited infiltration of neutrophils and alveolar collapse in the affected areas.

Upon necropsy, the liver appeared dark red, and the gallbladder was enlarged (Figure 1A). The lungs were congested and exhibited a dark red coloration, affecting the entire lobe, consistent with lobar pneumonia. Localized necrosis was observed in lungs (Figure 1B). Histopathological analysis revealed bilirubin crystals within bile ducts (Figure 1C), extensive hepatocyte necrosis centered around the central veins, disintegration of hepatic cords, congestion of hepatic sinusoids, and significant pigment deposition in hepatocyte cytoplasm (Figure 1D). The lungs showed extensive neutrophil infiltration and fibrinous exudate in the bronchioles and alveoli (Figures 1E,F), suggesting bacterial infection in the affected areas.

### 3.2 Bacterial isolation and phenotypic characterization

Colony morphology was uniform on blood agar. Single colonies exhibited swarming motility on BHI agar (Supplementary Figure 1A), a hallmark of *Proteus* species, characterized by concentric ring-like migration. Gram staining revealed red, non-spore-forming bacilli with rounded ends (Supplementary Figure 1B). Biochemical identification results (Supplementary Table 1) matched the profile of *P. mirabilis*.

### 3.3 Molecular identification via 16S rRNA sequencing

The 16S rRNA sequence of the isolate was 1,531 bp (Supplementary Figure 2A). The strain showed 99.73–99.93% homology with three strains of *P. mirabilis*, with the highest similarity (99.93%) to *P. mirabilis* ATCC 29906 (NR114419.1). When compared with two other strains of *P. mirabilis* isolated from Malayan pangolins (PP935660.1; ON076731.1), the homology was 99.63 and 98.70%, respectively. Homology with other *Proteus* species was slightly lower, ranging from 99.3 to 98.10%. The lowest homology was with *Cedecea lapagei*, at 93.80%. The phylogenetic tree shows that the isolated strain clusters closely with *Proteus mirabilis*, while there was a noticeable divergence from other species within the genus *Proteus* (Supplementary Figure 2B).

Based on morphological and 16S rRNA sequencing, the isolate was identified as *P. mirabilis* and designated PM2022.

### 3.4 Antimicrobial resistance profile of PM2022

To characterize the antibiotic resistance profile of the isolated strain, we conducted antimicrobial susceptibility testing using 35 commonly used antibiotics, as detailed in Table 1. Antimicrobial susceptibility testing revealed that PM2022 was susceptible to cefoxitin (cephalosporin), amikacin (aminoglycoside), imipenem and meropenem (carbapenems), while exhibiting intermediate susceptibility to ceftazidime (cephalosporin), neomycin (aminoglycoside) and azithromycin (macrolide). According to the Magiorakos et al. (26) criteria, a bacterial strain is classified as multidrug-resistant (MDR) when it exhibits non-susceptibility to at least one agent in three or more antimicrobial categories. PM2022 was classified as MDR as it resisted agents from five antimicrobial categories (penicillins, lincosamides, quinolones, tetracyclines, and chloramphenicol).

**Table 1:** The results of antibiotic sensitivity tests.

Class	Antibiotics	Zone/mm	Interpretation	Breakpoint/mm		
				R	I	S
Penicillins	PEN (1 μg)	0	R	≦ 25	28 ~ 26	≧ 29
	AMP (10 μg)	10	R	≦ 13	14 ~ 16	≧ 17
	OXA (1 μg)	0	R	≦ 10	11 ~ 12	≧ 13
	AMX (25 μg)	0	R	≦ 14	15 ~ 17	≧ 18
Cephalosporins	LEX (30 μg)	0	R	≦ 14	15 ~ 17	≧ 18
	FOX (30 μg)	20	S	≦ 14	15 ~ 17	≧ 18
	CFP (30 μg)	13	R	≦ 17	18 ~ 20	≧ 21
	TIO (30 μg)	8	R	≦ 19	20 ~ 22	≧ 23
	CAZ (30 μg)	19	I	≦ 17	18 ~ 20	≧ 21
	RAD (30 μg)	10	R	≦ 14	15 ~ 17	≧ 18
	CTX (30 μg)	12	R	≦ 19	20 ~ 22	≧ 23
	CRO (30 μg)	10	R	≦ 17	18 ~ 20	≧ 21
Carbapenems	MEN (10 μg)	26	S	≦ 19	20 ~ 22	≧ 23
	IPM (10 μg)	24	S	≦ 19	20 ~ 22	≧ 23
Aminoglycosides	GEN (10 μg)	8	R	≦ 12	13 ~ 14	≧ 15
	NEO (30 μg)	14	I	≦ 13	14 ~ 16	≧ 17
	KAN (30 μg)	0	R	≦ 14	15 ~ 17	≧ 18
	TOB (10 μg)	0	R	≦ 12	13 ~ 14	≧ 15
	AMK (30 μg)	19	S	≦ 14	15 ~ 16	≧ 17
Macrolides	ERY (15 μg)	0	R	≦ 19	20 ~ 22	≧ 23
	AZM (15 μg)	15	I	≦ 14	15 ~ 17	≧ 18
Lincosamides	CLI (2 μg)	0	R	≦ 17	18 ~ 20	≧ 21
Quinolones	NOR (10 μg)	13	R	≦ 13	14 ~ 16	≧ 17
	LEV (5 μg)	11	R	≦ 15	16 ~ 18	≧ 19
	ENR (10 μg)	7	R	≦ 18	19 ~ 21	≧ 22
Tetracyclines	TET (30 μg)	6	R	≦ 14	15 ~ 18	≧ 19
	DOX (30 μg)	0	R	≦ 12	13 ~ 15	≧ 16
Chloramphenicol	FFC (30 μg)	0	R	≦ 15	16 ~ 17	≧ 19
	CHL (30 μg)	0	R	≦ 14	15 ~ 17	≧ 18
Others	FUS (10 μg)	0	R	≦ 19	20 ~ 22	≧ 23
	SXT (20 μg)	6	R	≦ 12	13 ~ 15	≧ 16
	PB (300 μg)	0	R	≦ 9	10 ~ 11	≧ 12
	NIT (300 μg)	7	R	≦ 14	15 ~ 16	≧ 17
	RIF (5 μg)	8	R	≦ 16	17 ~ 19	≧ 20
	VAN (30 μg)	0	R	≦ 12	13 ~ 14	≧ 15

S, susceptible; I, intermediate; R, resistant. The breakpoints were based on the CLSI VET standards (2023 version).

### 3.5 Pathogenicity assessment in murine model

To assess the pathogenicity of the isolated strain, we determined its LD_50_ using the Karber method. The LD_50_ was calculated to be 1.45 × 10^9^ CFU/mL after 1 week, indicating high pathogenicity in mice (Supplementary Table 2). The livers of the deceased mice appeared dark red with pale yellow patches or pinpoint white spots on the surface; the lungs showed dark red spots on the surface. Histopathological examination revealed congestion in both the liver and lungs, and *P. mirabilis* was re-isolated.

### 3.6 Genomic analysis of resistance and virulence determinants

Based on the aforementioned LD_50_, antimicrobial susceptibility testing, and studies, whole-genome sequencing was performed on the isolated strain to elucidate the potential mechanisms underlying its multidrug resistance and high virulence. PM2022 genome is 3,961,270 bp in length, containing 3,650 coding genes, with an average gene length of 925.87 bp and a guanine-cytosine (GC) content of 38.83%.

Using the Comprehensive Antibiotic Resistance Database (CARD), it was found that PM2022 contains 26 ARGs, including *tet(J)*, *floR*, *SAT-2*, *TEM-1*, *CTX-M-65*, *PBP3*, *CRP*, *APH(3″)-Ib*, *catI*, *catB3*, *AAC(6′)-Ib-cr*, *AAC(3)-IV*, *aadA5, FosA3*, *OXA-1*, *APH(3′)-Ia*, *APH(6)-Id*, *dfrA17*, *dfrA1*, *APH(4)-Ia*, *arr-3*, *aadA*, *AAC(3)-IIa*, *adeF*, *Shigella flexneri* chloramphenicol acetyltransferase, and *Salmonella enterica gyrA* with a mutation conferring resistance to triclosan (Table 2). These genes may confer resistance to 15 classes of antimicrobial agents, including aminoglycosides, cephalosporins, penicillins, chloramphenicols, fluoroquinolones, carbapenems, diaminopyrimidines, monobactams, tetracyclines, nucleoside analogs, fosfomycins, rifamycins, triclosan, macrolides, and cephamycins.

**Table 2:** Drug resistance gene statistics.

Drug resistance class	Resistance genes
Aminoglycosides	*aadA, aadA5, AAC(3)-IIa, AAC(3)-IV, AAC(6′)-Ib-cr, APH(3′)-Ia, APH(3″)-Ib, APH(4)-Ia, APH(6)-Id*
Cephalosporins	*TEM-1, CTX-M-65, OXA-1, PBP3*
Penicillins	*CRP, TEM-1, OXA-1, PBP3*
Chloramphenicols	*floR, catI, catB3, CAT*
Fluoroquinolones	*CRP, adeF, AAC(6′)-Ib-cr*
Carbapenems	*TEM-1, PBP3, PBP3*
Diaminopyrimidines	*dfrA1, dfrA17*
Monobactams	*TEM-1, PBP3*
Tetracycline	*tet(J), adeF*
Nucleosides	*SAT-2*
Fosfomycins	*FosA3*
Rifamycins	*arr-3*
Triclosan	*gyrA*
Macrolides	*CRP*
Cephamycins	*PBP3*

Using the Virulence Factor Database (VFDB), a large number of virulence-related genes were predicted in the PM2022 genome. The virulence-related genes in PM2022 were statistically analyzed based on a comparison with the VFDB, with a consistency value of ≥ 80% (27). According to the pathogenic mechanisms (28), the annotated virulence factors from the VF database were classified into eight major categories: Adherence, Regulation, Motility, Nutritional/Metabolic factors, Effector delivery systems, Immune modulation, Biofilm, and Exotoxins. The PM2022 genome contains 20 virulence factors, comprising 59 virulence genes (Table 3).

**Table 3:** Virulence factor statistics.

Category	Virulence factor	Symbol
Adherence	PMF pili	PMI_RS09285, PMI_RS09280, PMI_RS09275, PMI_RS09270, PMI_RS09265
	MR/P	*mrpA*, *mrpB*, *mrpC*, *mrpD*, *mrpH*, *mrpI*, *mrpJ*, PMI_RS01295, PMI_RS01300, PMI_RS01305
	UCA	*ucaA*, PMI_RS02630, PMI_RS02635, PMI_RS02640, PMI_RS02645
	EF-Tu	*tufA*
Regulation	RcsAB	*rcsB*
	CsrA	*csrA*
	Fur	*fur*
Motility	Flagella	*flhC*, *flhD*, *fliA*, *fliG*, *fliP*, *flgG*, *cheW*, *cheY*
Nutritional/Metabolic factor	HmuR2	PMI_RS06920, PMI_RS06915, PMI_RS06910, PMI_RS06905, PMI_RS06900
	Proteobactin	PMI_RS01110, PMI_RS01115, PMI_RS01120, PMI_RS01125, PMI_RS01130, PMI_RS01135, PMI_RS01140, PMI_RS01145, PMI_RS01150, PMI_RS01155, PMI_RS01160
Effector delivery system	Pta	*pta*
	HpmA-HpmB	*hpmB*
		*hpmA*
	AipA	*aipA*
	T6SS	*hcp*
	T6SS-II	*clpV*
Immune modulation	Capsule	*gndA*
	LOS	*kdsA*
	Capsular polysaccharide	*rmlB*
Biofilm	Cah, AIDA-I type	*cah*
Exotoxin	ZapA	PMI_RS01355

## 4 Discussion

As an opportunistic pathogenic bacterium, *Proteus mirabilis* is widely present in the intestines of pangolins and poses a threat to their intestinal health, causing intestinal hemorrhage, mucosal damage, diarrhea and even death (19, 21, 29). Additionally, *P. mirabilis* can affect the respiratory tract of pangolins. Liu et al. (21) isolated *P. mirabilis* from the lungs of a deceased wild Malayan pangolin, which exhibited tracheal purulent secretions and pulmonary mucosal edema and hemorrhage. Zhang et al. (29) isolated *P. mirabilis* and *Klebsiella pneumoniae* from the nasal cavities of Malayan pangolins with sneezing and dyspnea. However, the antibiotic resistance profiles, resistance genes, and virulence gene characteristics of *P. mirabilis* isolated from pangolins are not well understood. This study isolated and identified *P. mirabilis* (PM2022) from a Malayan pangolin, analyzing its antibiotic resistance, ARGs, pathogenicity and virulence genes.

*Proteus mirabilis*, as a Gram-negative bacterium, is characterized by peritrichous flagella, conferring strong motility (9). This enables rapid colonization on surfaces like agar. Therefore, the colony characteristics of PM2022 on BHI agar and its morphology after Gram staining are similar to those observed in previous studies on *P. mirabilis* (30, 31). Biochemical identification matched *Proteus* spp. profiles, and 16S rRNA gene sequencing and phylogenetic analysis confirmed PM2022 as *P. mirabilis*.

Excluding carbapenems (meropenem and imipenem) (32), which are strictly prohibited in animals, PM2022 was only susceptible to cefoxitin (cephalosporin) and amikacin (aminoglycoside). This resistance pattern resembles *P. mirabilis* isolates YN2018 (Malayan pangolin source) (21) and FJ/Tiger01 (South China tiger source) (16), but PM2022 exhibited broader resistance and was identified as a MDR. The antibiotic resistance of *P. mirabilis* is becoming increasingly severe (33), with many studies reporting multidrug resistance in isolates from various sources (27, 34, 35). This poses a significant challenge for clinical treatment in animals.

ARGs are key drivers of bacterial resistance (36, 37), with strong genotype–phenotype correlations (38). CARD annotation identified 26 ARGs in PM2022, conferring resistance through mechanisms such as antibiotic efflux, antibiotic inactivation, and antibiotic target alteration (39). Most ARGs mediated antibiotic inactivation, including *SAT-2*, *TEM-1*, *CTX-M-65*, *aadA*, *AAC(3)-IIa*, *APH(3′)-Ia* and *FosA3*. Many ARGs for penicillins, fluoroquinolones, chloramphenicols, and tetracyclines were detected, explaining PM2022’ s phenotypic resistance. Although aminoglycoside-resistance genes were abundant, PM2022 remained susceptible to amikacin, suggesting that there may be other resistance mechanisms to aminoglycoside antibiotics. Genotypic analysis revealed that both PM2022 and YN2018 carried resistance genes to aminoglycosides, sulfonamides, and tetracyclines (21). Notably, PM2022 also possessed *β*-lactam and quinolone resistance determinants, which may contribute to its stronger and broader antimicrobial resistance profile compared to YN2018. The antibiotic resistance of *P. mirabilis* is continuously increasing (9), veterinarians must prudently select antibiotics to prevent XDR strains in captive pangolins. The antimicrobial susceptibility profile suggests that cefoxitin and amikacin may be considered as potential therapeutic options. Based on the ARGs identified and previous studies (21, 29), the use of penicillins, fluoroquinolones, and tetracyclines should be avoided in the treatment of *P. mirabilis* infections of pangolins. Additionally, while studying the resistance mechanisms of *P. mirabilis*, it is crucial to explore new therapeutic approaches, which have significant implications for both animal healthcare and public health.

Compared to other *P. mirabilis* isolates, such as CC15031 (canine source, LD_50_ = 0.57 × 10^6^ CFU/mL) (27), YL1 (porcine source, LD_50_ = 1.46 × 10^7^ CFU/mL) (40), SKPM 2 (sika deer source, LD_50_ = 3.98 × 10^7^ CFU/mL) (41), and PM19 (peacock source, LD_50_ = 6.19 × 10^6^ CFU/mL) (34), PM2022 exhibited relatively high pathogenicity in mice (LD_50_ = 1.45 × 10^9^ CFU/mL). Further annotation of PM2022 using the VFDB revealed that PM2022 contains eight categories of virulence factors (28). Genes related to nutritional and metabolic factors (Proteobactin, HmuR2) and adherence (PMF pili, MR/P, UCA) were detected at higher rates than other genes. *P. mirabilis* possesses excellent bacterial motility. Nine virulence factors associated with flagellum formation and 12 virulence factors associated with hyphal formation were identified in CC15031 (27). In PM2022, eight flagella-related virulence genes (*flhC*, *flhD*, *fliA*, *fliG*, *fliP*, *flgG*, *cheW*, *cheY*) were identified, which may contribute to enhanced motility and virulence of the bacterium. The Type VI Secretion System (T6SS) detected in PM2022 is a bacterial weapon widely distributed in Gram-negative bacteria, which can help kill competing bacteria or host cells and plays a crucial role in bacterial activities (42). This secretion system is increasingly being studied due to its association with pathogenesis and microbial competition (43). In terms of toxins, PM2022 also harbors Proteus toxic agglutinin (Pta), hemolysins (HpmA-HpmB), and the metalloprotease ZapA. Pta acts both as a bacterial autoagglutinin and a toxin to host cells, causing host cell membrane damage, actin depolymerization, and ultimately cell lysis (10). Hemolysins can lyse red blood cells and nucleated cells (44). ZapA degrades host immunoglobulins, complement proteins, extracellular matrix, cytoskeletal proteins, and even antimicrobial peptides. It also serves as an important mechanism for immune evasion in *P. mirabilis* (45). Based on nacropsy findings, murine experiments, genomic data and previous studies (21, 29), the flagella of *P. mirabilis* may serve as key virulence factors contributing to multi-organ infection (20), with pangolin strains targeting the intestines, liver, and lungs. The virulence factors of PM2022 can degrade the host’s tissue structures, promoting bacterial invasion and dissemination. Their effects were particularly evident in the pathological changes observed in the liver and lungs, manifested as hepatocyte necrosis, disintegration of hepatic cords, and alveolar collapse. The infection and inflammation in the respiratory system further impaired gas exchange, potentially leading to respiratory failure and contributing to the death of the pangolin.

In this study, we isolated a strain of *Proteus mirabilis* (PM2022) from the lungs exhibiting lobar pneumonia and the severely necrotic liver of a deceased Malayan pangolin. This strain demonstrated a multidrug-resistant phenotype with a narrow susceptibility profile, and harbored 26 ARGs, including *CTX-M-65*. It also carried 20 virulence factors and demonstrated high pathogenicity in murine models. Experimental and genetic evidence suggests that this bacterium likely targets the lungs and liver as primary infection sites in pangolins. For clinical treatment, cefoxitin and amikacin are potential treatments, while penicillins and fluoroquinolones should be avoided. This study provides the first detailed analysis of *P. mirabilis* in pangolins, offering evidence-based treatment recommendations to enhance rescue outcomes and support conservation of this critically endangered species.

## Data Availability

The complete nucleotide sequence of the *Proteus mirabilis* isolate in this study has been uploaded to GenBank under the BioProject number PRJNA1201381 (https://www.ncbi.nlm.nih.gov/bioproject/PRJNA1201381).
