# Supplementary material for: Multidrug-resistant Proteus mirabilis in a critically endangered Malayan pangolin: clinical and genomic insights
Source: Front Vet Sci. 2025 Apr 30;12:1552499. doi: 10.3389/fvets.2025.1552499 (PMC12075528; doi:10.3389/fvets.2025.1552499)
Supplement: Supplementary file 1 [file Data_Sheet_1.docx]

Supplementary Material

## Supplementary Figures


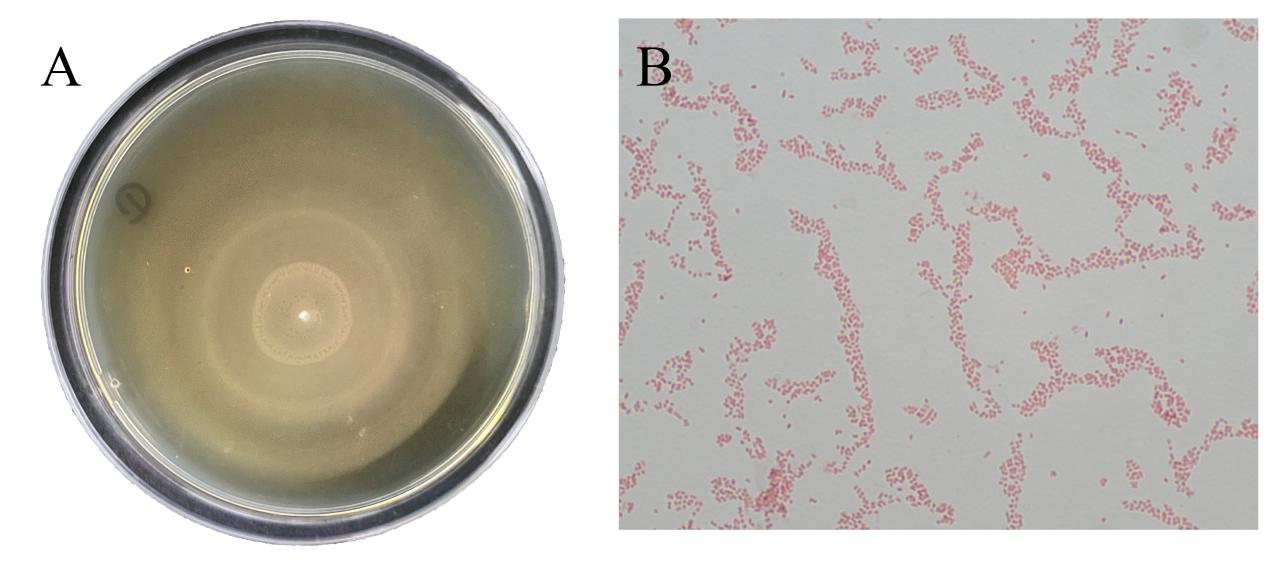


**Supplementary Figure 1. (A)** The single colony morphology on BHI agar exhibited swarming motility on BHI agar. **(B)** Microscopic examination after Gram staining revealed that the isolated strain appears as red, non-spore-forming, short rods with blunt ends (100 ×).


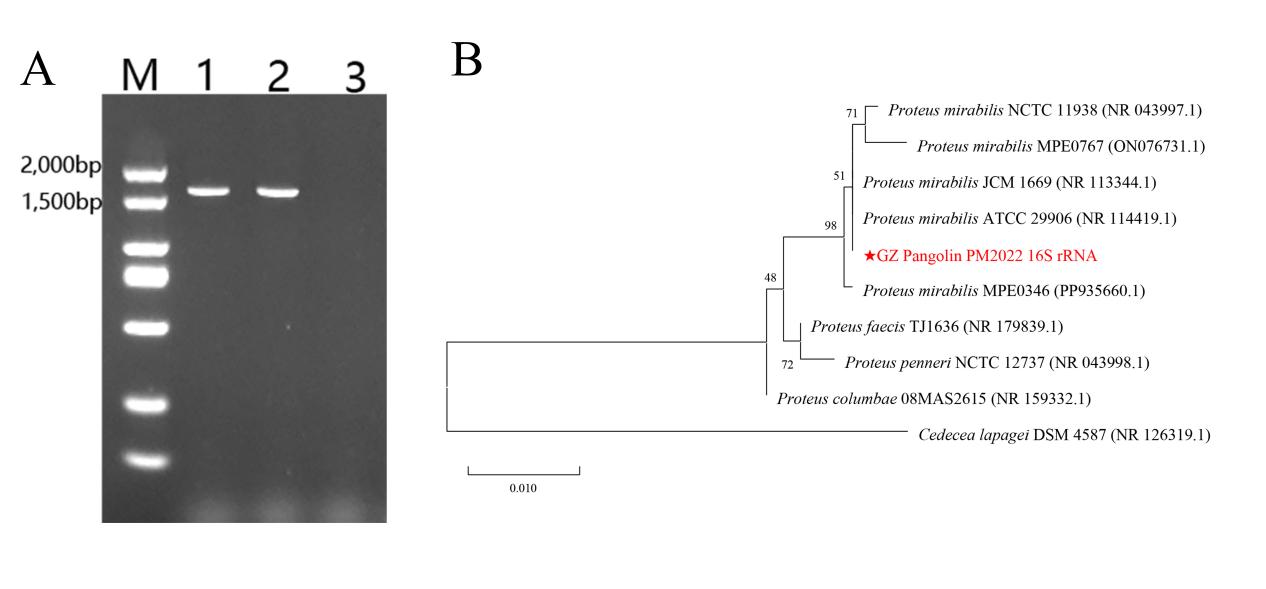


**Supplementary Figure 2. (A)** Gel electrophoresis results of the 16S rRNA amplification products of the isolate indicated a sequence length of about 1,500 bp. M: DL2,000 DNA Marker; 1-2: Amplification products of the isolate; 3: Negative control. **(B)** A phylogenetic tree constructed using the maximum likelihood method based on the 16S rRNA sequences showed that PM2022 clusters with *P. mirabilis*, while being distinct from other species of the genus *Proteus*.

## Supplementary Tables

**Supplementary Table 1.** The results of biochemical test

| **Biochemical Tests** | **Result** |
| --- | --- |
| Phenylalanine deaminase | + |
| Simmons' citrate | + |
| Tryptophan broth | - |
| Gelatin | + |
| Ornithine decarboxylase broth | + |
| Amino acid decarboxylase control | + |
| Urease | + |
| Xylose | + |
| Maltose | - |
| Lipase | - |
| Mannitol | - |
| Salicin | - |
| Esculin hydrolysis | - |

**Supplementary Table 2.** The mortality of mice in pathogenicity test

| **Group** | **Suspension Concentration (CFU/mL)** | **Number of Death** |
| --- | --- | --- |
| 1 | 2.9×10^7^ | 0/10 |
| 2 | 2.9×10^8^ | 0/10 |
| 3 | 2.9×10^9^ | 8/10 |
| 4 | 2.9×10^10^ | 10/10 |
| 5 | 2.9×10^11^ | 10/10 |
| Control | 0 | 0/10 |
